# Rural-Urban Differences in the Long-Term Care of the Disabled Elderly in China

**DOI:** 10.1371/journal.pone.0079955

**Published:** 2013-11-05

**Authors:** Mei Li, Yang Zhang, Zhenyu Zhang, Ying Zhang, Litao Zhou, Kun Chen

**Affiliations:** 1 Department of Epidemiology and Health Statistics, School of Public Health, Zhejiang University, Hangzhou, China; 2 Clinical Evaluation Analysis Center, Traditional Chinese Medicine Hospital of Zhejiang, Hangzhou, China; 3 Zhejiang Hospital, Hangzhou, China; University of Glasgow, United Kingdom

## Abstract

**Background:**

In China, the rapid rate of population aging and changes in the prevalence of disability among elderly people could have significant effects on the demand for long-term care. This study aims to describe the urban-rural differences in use and cost of long-term care of the disabled elderly and to explore potential influencing factors.

**Methods:**

This study uses data from a cross-sectional survey and a qualitative investigation conducted in Zhejiang province in 2012. The participants were 826 individuals over 60 years of age, who had been bedridden or suffered from dementia for more than 6 months. A generalized linear model and two-part regression model were applied to estimate costs, with adjustment of covariates.

**Results:**

Pensions provide the main source of income for urban elderly, while the principal income source for rural elderly is their family. Urban residents spend more on all services than do rural residents. Those who are married spend less on daily supplies and formal care than the unmarried do. Age, incapacitation time, comorbidity number, level of income, and bedridden status influence spending on medical care (β=-0.0316, -0.0206, 0.1882, 0.3444, and -0.4281, respectively), but the cost does not increase as the elderly grow older. Urban residents, the married, and those with a higher income level tend to spend more on medical equipment. Urban residence and living status are the two significant factors that affect spending on personal hygiene products.

**Conclusions:**

The use of long-term care services varies by living area. Long-term care of the disabled elderly imposes a substantial burden on families. Our study revealed that informal care involves huge opportunity costs to the caregivers. Chinese policy makers need to promote community care and long-term care insurance to relieve the burden of families of disabled elderly, and particular attention should be given to the rural elderly.

## Introduction

In China, the number of persons aged 60 and over reached 180 million in 2010, representing 13% of the total population [1]. According to projections, China’s elderly population will reach 400 million by 2040 and 437 million by 2051; the number of elderly is increasing at a rate of 5.96 million per year because of decreasing mortality rates and longer life expectancy [2,3]. This demographic trend will lead to new patterns of growing morbidity among the elderly, meaning an increase in the proportion of people who suffer from mental or physical disabilities that prevent them from performing everyday living activities[4]. An estimated 33 million Chinese over the age of 60 have such limitations, and almost a third of them are dependent on others for assistance [5].

 Changes in the prevalence of disability among elderly people could have significant effects on the demand for assistance, and a corresponding growth in demand for social care services is both expected and inevitable [1]. In planning for the country’s future long-term care (LTC) provision, it is important to understand what factors influence and is influenced by the cost of LTC services. This information will not only help policymakers to understand the health needs and expenditure burden of elderly people and their families, but will also be useful in developing the LTC insurance system. Many studies in Asian and Western countries have shown that several factors, such as age, gender, marriage, medical complications, and living with children, may affect the use and cost of LTC [4,6,7]. 

 As in other Asian countries, family values in China are highly regarded and embedded in a cultural tradition of filial piety [8]. In rural China, financial support for the elderly remains the responsibility of adult children, and is even codified into laws governing the family [9]. However, modernization, demographic shifts, and the massive outmigration of young people to cities for work are eroding this tradition, raising concerns that the supportive functions of families have been weakened [10,11]. In cities, meanwhile, the emerging “4:2:1” family structure (four grandparents, two adult children, and one grandchild) is emblematic of the problem: two adults have to take care of four parents and raise one child [12]. Either way, most older people prefer to live in their own home for as long as possible, making the issue even more pressing, while the resources and infrastructures for aged care are extremely limited; public support systems, such as pensions and health insurance, are underdeveloped or absent [13]; and community-based or institutional-care services remain largely nonexistent, except in a few major urban centers like Shanghai [14]. 

 Most of the care within an older person’s home is informal care provided by family members, although this informal care may be supplemented by formal services provided at home by paid caregivers [13]. Because LTC is a new concept in China, insufficient data exist on the spending and use of the services and on the extent and nature of unpaid informal care that people are receiving; nor has the cost to families in economic, social, and material terms been assessed. This paper intends to fill this gap by investigating a sample of rural and urban elderly in Zhejiang province, China, and answering the following questions:

1. What is the average spending on LTC services for the elderly living in urban and rural areas?
2. What factors (location, gender, age, living arrangements, etc.) influence the use and cost of LTC services?

## Materials and Methods

### Ethics statement

All participants were informed about the content of the questionnaire and the aim of the study before the survey. For disabled people who had a compromised capacity to consent, their next of kin or caregiver consented on their behalf. The study was approved by the institutional review boards of Zhejiang University, Hangzhou, China. Written, informed consent was collected from all participating subjects. 

### Study sample

A cross-sectional survey was carried out through face-to-face interviews in Zhejiang province between March and July 2012. Zhejiang province, located in the Yangzi River Delta, has faced serious problems caused by a rapidly aging population. According to the 2008 National Sample Survey on Population Changes, people aged 65 and over accounted for 10.6% of Zhejiang’s population [15]. A multistage, random cluster sampling procedure was applied to produce the samples. Cities in the provinces were stratified by income (low, middle, and high, according to their gross domestic product [GDP] in 2011) and location, and four cities were chosen at random out of 11. One urban district and one rural county in each city were then randomly selected from a list stratified by elderly population size, and one block was randomly chosen from each district and county; all people in that block meeting the inclusion criteria were included in the study, using the full name list provided by Zhejiang Provincial Working Committee on Aging. People were selected if they were 60 years old or over and bedridden or suffering from dementia (having been diagnosed by doctors) for more than 6 months. People were excluded if patients or their caregivers refused to participant in the survey or if the patients or their caregivers were unable to communicate with the investigator. In this paper, subjects are described as “disabled elderly” if they have been bedridden or suffered from dementia for over 6 months and are aged over 60. The minimum sample size was 800, assuming that each cluster was selected with equal probability [16]. The interviewers, who were trained master’s degree students, had had appropriate training in interview techniques and survey methodology. Out of 991 interviewed persons, 926 agreed to participate in the survey, a response rate of more than 93%. Of these, 54 participants were excluded because their period of disability was shorter than 6 months, 24 participants were excluded because there was no information about their dementia or bedridden status, and 20 participants were excluded because they were neither bedridden nor suffering from dementia. The final study population consisted of 826 participants, and the adjusted response rate was 83.35%. 

 The structured questionnaire was developed by the public health department of Zhejiang University, and pilot testing was conducted with a convenience sample of 30 respondents drawn from the selected population. The questionnaire mainly comprised questions about patients’ socio-demographic characteristics (age, gender, place of residence, education level, marital status, living arrangement, source of income), disability status (bedridden, suffering from dementia, other diseases), care provider’s characteristics (age, gender, time spent on caring every month), purchase/rent of medical equipment (wheelchair, canes, bathing equipment, etc.), purchase of personal hygiene products (diapers, pads, urine collection devices, etc.), expenditure for medical care (out-of-pocket, covered by insurance), cost of caregivers each month, cost of daily supplies, and patients’ economic circumstances (income, source of income, insurance coverage). To control for quality of the data, data management activities were performed. At the end of each interview, completeness of the data collection was checked by the researcher. Double data entry was performed by different data enterers into Epidata 3.2.

### Statistical analysis

The cost of care per patient for 1 month was equivalent to all direct and indirect costs incurred by the patient, family members, care providers, and insurer during this period [17]. All questions relating to time spent on different caregiver activities were based on the number of hours spent on these activities on a typical day.

 Indirect costs for patients and families included the economic value of informal care. Informal care, which was usually offered by family members without payment (unpaid caregivers), is not a free resource. The provision of informal care entails opportunity costs in various forms, such as the labor costs of family caregivers taking care of patients at home, investment of energy, sacrifice of paid work time or leisure time, and possibly a reduction in social contacts [6]. The loss of earnings by patients was not included in the total cost, on the assumption that it would have little impact because they were unable to work because of disability. In this paper, we adopt the opportunity cost method, which values the time investment in informal care by assessing the net market wage rate of each caregiver and the time spent on care tasks [18]. In this study, the local average hourly wage of a formal caregiver was used for the labor cost of family caregivers. 

 Direct costs incurred by patients were the out-of-pocket expenditures for medical care, including physician services and prescription drugs, purchases/rent of medical equipment or personal hygiene products, spending on daily supplies, and the cost of formal care services. In our study, formal care services were defined as those provided by paid caregivers. For medical equipment (such as wheelchairs, canes, bathing equipment, etc.), if the patients purchased the tool, we set a use limit of 5 years to evaluate the cost for each year. Patients and caregivers were also encouraged to list and report any additional care-related expenditure. 

 We applied a generalized linear model (using gamma link because all expenditure is positive) to estimate the informal care costs, medical costs, and cost of daily supplies, and we identified potential impact factors, such as age, gender, location, etc. For expenditure on formal services, medical equipment, and personal hygiene products, zero costs were observed with sufficient frequency to warrant the application of a two-part model to estimate the adjusted expenditure and the potential factors influencing costs [19]. The expected value of expenditure y conditional on the covariates x is 

E(Y|X)=P(Y>0|X)E(Y|Y>0,X)

In part I, y=0 if no cost is incurred and y=1 if cost is incurred; the probability of positive expenditures Pr(Y>0| X) is assumed to be governed by a parametric binary probability model. Here, we use logistic regression and a maximum likelihood function to estimate the log odds ratio: log(P_i_/1-P_i_)=α+βX. In part II, a generalized linear model with gamma link was applied to estimate the amount of expenditure and involved only those observations with non-zero cost (conditional cost regression) [20]. In both parts, we include covariates, age, sex, education level, marriage status, living arrangements, bedridden or dementia, incapacitation time, number of comorbidities, and income level. The effect of a covariate is considered to be significant if the effects of it in both parts have the same sign and at least one of the two p-values is significant [21]. All analyses were performed with the software package SAS, version 9.2.

## Results

Characteristics of the study sample by rural and urban areas are shown in Table 1. The average age for the subjects is 81 years, and 59.9 % are women. The elderly living in urban areas are more likely to be single (χ^2^ = 4.526, p = 0.033), living alone (χ^2^ = 42.83, p = 0.000), educated to a higher level (χ^2^ =137, p<0.001), have a higher income (χ^2^ = 45.51, p<0.001), and be incapacitated for longer (p = 0.029) than the rural elderly. There are no significant differences between rural and urban subjects in terms of age and gender. Hypertension, cardiovascular disease, and diabetes mellitus are the most common comorbid conditions in the elderly, and they are all more prominent in the urban elderly. 

**Table 1:** Baseline characteristics of the studied sample (N=826).*

	**Rural**	**Urban**	**p-value**
N	435	391	
**Mean age (SD)**	82(7.1)	80(8.6)	0.593
60–69	61	43	0.133
70–79	127	115	
80–89	176	184	
>90	71	49	
**Gender**			0.298
Male	167	164	
Female	268	227	
**Marital status**			0.033
Married	170	195	
Single^+^	228	195	
**Education level**			<0.001
Primary school and below	421	254	
Middle school	12	114	
College and above	0	21	
**Living arrangements**			<0.001
Lives alone	82	22	
Lives with others	353	369	
**Incapacitation time**	5.14+6.03	6.19+6.66	0.029
**Comorbidity, n (%)**			<0.001
Hypertension	169(38.9)	241(61.6)	
Cardiovascular	61(14)	116(29.7)	
Cerebrovascular	89(20.5)	139(35.5)	
Diabetes mellitus	36(8.3)	94(24.0)	
**Yearly personal income (Yuan)**			<0.001
<=8000	322	40	
8000–30000	72	242	
>30000	2	106	

* Data were missing for some respondents for the following characteristics: marital status (38), educational level (4), yearly personal income (42).

^+^ Including unmarried, divorced, separated, and widowed.

Table 2 presents the unadjusted mean cost and use of services by location and gender. Time spent on formal care is estimated at 533 hours per month for rural residents and 612 hours for urban residents, on average. For rural men, the average spending on paid caregivers per month is 4020 RMB, which is higher than that for urban men (2,370 RMB), but the service use in rural areas is much less than in urban areas (5.9% vs. 36.9%). The average cost of informal care is estimated at 1470 RMB for a rural family and 2991 RMB for an urban family when informal care time is valued using the opportunity cost method. The primary caregiver in rural areas is the son, whereas the primary caregiver in urban areas is the spouse. Most of the informal caregivers are female (58.8% in rural areas and 52.2% in urban areas). The spending on medical equipment is low for the rural elderly, at only 4.1 RMB per month, on average. The use of personal hygiene products by the urban elderly is almost triple that of the rural elderly (46.8% vs. 18.9%). Insurance covers most of the medical care costs (1637.6 RMB out of 2322.9 RMB) for urban residents, whereas family members are the primary payers for the rural elderly (547.5 RMB out of 828.8 RMB); the use of medical care by men is higher than that by women in both urban and rural areas. 

**Table 2:** Monthly use of and spending on services for rural and urban residents.

	**Rural**			**Urban**		
**Formal care**	Average	Male	Female	Average	Male	Female
Costs	2473.0	4020	1613	2320.0	2370	2290
Hours	533	533	533	612	585	627
Service use (%)	5.9	5.5	6.1	36.9	31.7	40.9
**Informal care**						
Cost	1319.0	1470	1212	2991.0	3248	2797
Hours	378	420	353	613	657	575
Service use (%)	91.8	80.8	92.4	87.7	90.2	85.9
Relationship to recipient (%)						
Spouse	24.3	37.6	16.1	33.1	47.5	20.5
Son	33.1	29.7	35.1	28.1	26.9	29.1
Daughter	21.9	11.8	18.2	28.1	20.7	34.5
Daughter in law	15.8	17.5	24.7	8.7	3.7	12.9
Other	4.9	3.4	5.9	2	1.2	3
Female caregiver (%)	52.2	66.8	43	58.8	71.9	54.5
**Medical equipment**						
Total cost	4.1	3.7	4.6	30.2	24.8	35
Service use (%)	35.6	39.9	32.8	71.8	81	65.2
**Personal hygiene products**						
Total cost	175.8	168.7	180.8	275.9	224.1	319.3
Service use (%)	18.9	20.2	18.1	46.8	51.5	43.5
**Medical care**						
Total cost	828.8	1811.6	677.6	2133.6	1448.8	793.5
Service use (%)	87.9	97.5	88.7	97.5	97.4	89.9
Private payment (self)	231.4	273.2	199.7	461.4	624.6	341.2
Private payment (children)	316.1	311.3	318.5	351.1	279.9	391.5
Insurance company	396.1	488.3	330.5	1637.6	2194.8	1232.2
**Daily supplies**	456.2	486.0	437.6	1482.6	1667.6	1350.2

Table 3 illustrates the source of participants’ income and the primary payers for services. The main source of income for the urban elderly is the pension, while the principal income source of the rural elderly is family support; less than 3% of rural elderly said that a pension is their principal source of income. For formal care, children are the primary payer for rural residents, whereas the urban elderly and their spouses are their primary payers. Elderly men and their spouses pay for most of medical equipment and personal hygiene products, whereas elderly women are more dependent on their children for these.

**Table 3:** Source of support and primary payer of services for disabled elderly (percentage).

	**Rural**		**Urban**	
	male	female	male	female
**Source of support**				
Pensions	3	1.1	93.4	73.9
Family Support	75.7	81.5	18.7	32.6
Property Income	0.0	0.4	1.8	0.9
Financial Aid	10.9	13.2	1.8	2.6
Insurance	39.4	35.5	41.9	58.1
Subsidy	33.3	37.4	15.7	21.7
**Formal care**				
Spouse or Self	20.0	26.7	81.1	54.9
Children	70.0	73.3	18.9	41.8
Others	10.0	0.0	0.0	3.3
**Medical equipment**				
Spouse or Self	49.2	39.5	59	36.4
Children	38.5	46.5	33.6	53.6
Government	10.8	5.8	3.7	5.3
Others	1.5	8.2	3.7	4.6
**Personal hygiene products**				
Spouse or Self	57.9	37.3	68.4	39.6
Children	43.0	61.6	31.2	60.1

Table 4 describes the model-based cost estimates, adjusting for covariates (location, age, gender, marital status, education level, living arrangements, incapacitation time, type of disability, comorbidity, and income) using a generalized linear model. The urban elderly spend more on informal care services, medical services, and daily supplies than the rural elderly do. Income level significantly affects spending on informal care and medical services. The married elderly spend less on daily supplies, but the estimated informal value is higher for married elderly than for unmarried elderly. The age, incapacitation time, extent of comorbidity, and bedridden status influence spending on medical care, but the cost does not increase as participants’ age, although those who live with others spend more on both daily supplies and informal care. Education level affects daily supplies significantly.

**Table 4:** Factors associated with the use of and spending on services using generalized linear model.

	**Informal care**		**Medical**		**Daily supplies**	
	Impact	Impact	Impact	Impact	Impact	p-value
Urban	0.5809	<.0001	0.4925	<.0001	0.7571	<.0001
Female	-0.0826	0.2296	-0.0795	0.4106	-0.0636	0.1560
Age	0.0071	0.0779	-0.0316	<.0001	-0.0028	0.2904
Married	0.2013	0.0112	0.0560	0.5924	-0.1690	0.0007
Middle school	0.0384	0.6833	0.0397	0.7617	0.3735	<.0001
College or above	0.1615	0.4098	0.4116	0.1194	0.2524	0.0468
Lives with someone	0.3144	0.0010	0.0569	0.6481	0.1250	0.0339
Incapacitation time	0.0080	0.0719	-0.0206	<.0001	-0.0006	0.8318
Comorbidity	0.0098	0.6350	0.1882	<.0001	0.0559	<.0001
Dementia	-0.1317	0.0337	-0.4281	<.0001	-0.0006	0.9876
Medium income	0.1465	0.0659	0.3362	0.0020	0.3410	<.0001
High income	0.1044	0.3837	0.3444	0.0422	0.4000	<.0001

Table 5 summarizes the model-based cost estimates for formal care, medical equipment, and personal hygiene products adjusting for covariates using a two-part model because of low service use. The urban elderly use all these three services more often than do the rural elderly. The association between marriage and the cost per user of formal care is negative; use of formal care by the urban and high-income elderly is significantly higher than in the rural and low-income elderly. The urban, married, and higher-income elderly tend to spend significantly more on medical equipment, but the effect of age and incapacitation time is negative. Urban residence and living status are the two main factors affecting spending on personal hygiene products.

**Table 5:** Factors associated with the use and spending of services using two-part model.

	**Formal care**							**Medical equipment**					**Personal hygiene products**				
	Probability of use				Cost per user			Probability of use			Cost per user		Probability of use		Cost per user		
	Impact	p-value			Impact	p-value		Impact	p-value		Impact	p-value	Impact	p-value	Impact	p-value	
Urban	1.5947		<.0001		0.2071		0.0879	0.4920	0.0380		1.2671	<.0001	0.8818	0.0003	0.4150	0.0043
Female	0.4932		0.0402		-0.1847		0.0173	-0.1736	0.3451		0.629	0.0001	-0.2082	0.2648	0.2340	0.0633
Age	0.0051		0.7046		-0.0021		0.6559	-0.0063	0.5697		-0.0283	0.0018	0.0022	0.8445	-0.0127	0.0585
Married	-0.6373		0.0110		-0.1837		0.0181	0.1064	0.6097		0.5915	0.0005	0.0489	0.8132	-0.2865	0.0267
Middle school	0.6234		0.0164		-0.0598		0.4837	-0.1167	0.6457		0.0347	0.8675	-0.1561	0.5136	0.1326	0.3555
College or above	-0.6059		0.7646		-0.0430		0.8068	0.9412	0.1657		-0.2376	0.4927	-0.6522	0.1964	.01325	0.6862
Lives with someone	-0.2118		0.4848		0.1274		0.2388	-0.4078	0.0932		0.0503	0.8115	0.1087	0.6741	0.3870	0.0334
Incapacitation time	-0.0030		0.8260		0.0135		0.0123	-0.0342	0.0130		-0.0298	0.0240	0.0111	0.2381	-0.0103	0.1765
Comorbidity	0.0478		0.4331		0.0210		0.3415	0.2928	<.0001		-0.0442	0.2872	0.1186	0.0286	-0.0248	0.4921
Dementia	-0.0087		0.9651		0.0983		0.1703	-0.6064	0.0004		0.4650	0.0019	-0.0678	0.6871	-0.0501	0.6606
Medium income	0.0543		0.8560		0.1686		0.1142	0.8136	0.0003		0.5509	0.0135	0.4219	0.0716	-0.0259	0.8554
High income	1.0784		0.0044		0.1520		0.2120	1.4943	<.0001		0.8414	0.0043	0.3229	0.3224	-0.0225	0.9063

## Discussion

In this study, we assess the costs of taking care of disabled elderly individuals living in urban or rural areas in China and provide some insights into the determinants of care use and costs. 

 The rapid growth in number of disabled elderly is one of the most difficult problems to be solved as China’s population ages. The demand for LTC is likely to increase, formal LTC systems are emerging but remain in the preliminary stages of development [22], and the rate of use for health services at home among the disabled elderly is relatively low, especially in rural areas. Our study showed that only 5.9% of the disabled elderly in rural areas reported receiving any formal care, whereas the rate in urban areas is 36.9%, but average spending on formal care is higher in rural areas than in urban areas, especially for men. The difference may be explained by socioeconomic status because level of income significantly determines the use of services after adjusting for other covariates. In rural areas, only wealthy families can afford formal care. 

 Informal care by spouse or children is still the most common source of care for elderly people in China. Consistent with many studies, we confirmed the high utilization rate (around 90%) and costs of informal caring for the elderly [6,23]. Most informal care is provided by females, 47.5% by spouses and 28.1% by daughters in urban areas, compared to 37.6% by spouses and 11.8% by daughters in rural areas. Sons also took some responsibility for provision of care, especially in rural areas; most of the elderly in rural areas have more than one child, and their son will take a turn in caring for them. 

 Using the opportunity cost method, the estimated value of informal care in urban areas is 2991 RMB per month, while the cost is 1319 RMB per month in rural areas. A national survey carried out in 2006 showed that 7.4% of the population in urban areas were disabled elderly and 8.5% in rural areas [24]. If these individual received informal care similar to those in this study, after translating the results into a nationwide average, the estimated economic value of informal care provided in China is equal to 

3.85 billion RMB for the whole country per month, or an annual opportunity cost of 46.3 billion RMB. In the United States, informal assistance provided to the elderly represents about $257 billion [25]. In France, informal assistance provided to the elderly living at home was estimated to run to more than €6 billion [7]. National estimates of the costs of informal care in the United Kingdom vary widely, ranging between €34 billion and €57 billion per year [26]. Ignoring the monetary value of informal care would result in a huge underestimation of the cost of LTC. Policymakers could use this new information in the decision-making process when allocating funds for health care.

 Some previous studies have shown that living alone was positively associated with the use of formal care by the disabled elderly [4,27], whereas other research found that older people living alone were less likely to receive either formal or informal help [28]. In our study, the disabled elderly living alone were less likely to receive informal help than those living with spouses or children, but the relationship with formal care is unclear. The married elderly are more likely to get informal care and less likely to use formal care services. This is consistent with the fact that single people have fewer resources in terms of informal support and therefore are more likely to rely on formal services [21].

 Income level is positively associated with use of formal care, use of medical equipment, spending on medical care, and spending on daily supplies. Our study showed that support from children is the most important income source in rural areas and that children are the primary payers for formal care and auxiliary tools in rural areas, whereas the urban elderly or their spouses tend to pay for services by themselves or their spouses. This result is consisted with a survey that was carried out on 1% of the Chinese population [9], which found individuals with higher incomes have a lower cost of informal care use confirms the belief that elderly people with a higher income tend to replace informal care with private formal care.

 Expenditure on LTC usually rises with age [29]. A study in Germany showed the significant impact of age on the cost of statutory health insurance, LTC insurance, and formal care [21]. In our study, the effect of age is negative, which may be explained by the values of traditional Chinese culture, according to which, when people become bedridden or suffer from dementia, their relatives initially tend to resort to expensive medicine to help the patient recover; as time goes by, however, they may give up or just use basic medicines to maintain the patient’s life. 

 A national survey showed that between 1980 and 2002, China’s total health expenditure rose from 3.2% to 5.4% of GDP, while government health spending as a percentage of GDP declined from 1.1% to 0.8%, which may suggest that the growing financial burden of health care has largely fallen on individuals [30]. In our study, the average monthly out-of-pocket expense is 557 RMB. Given that over 80% of rural elderly earn less than 667 RMB per month, the need to cover high out-of-pocket medical costs imposes a heavy burden, and low-income families in particular may find themselves driven into poverty because of unaffordable medical expenses.

 We are aware of several limitations to this study that should be acknowledged. First, this is not a longitudinal but a cross-sectional survey, so the associations found in the study should be interpreted cautiously. Second, the self-reported data were used as the single source of information, which may be subject to recall bias, although most of the questions involved only recent months. Third, some useful variables were unavailable or not optimally coded. Comorbidity was adjusted for, but the severity of illness and functional status were not coded, so this adjustment was likely to be incomplete. In addition, the severity of disability was not recorded and so was not taken into account in the regression model. Finally, because information about occupation and service details for caregivers was unavailable, we use the average local formal care wage to estimate the value of informal care; since over half of all caregivers were women, that informal care cost may have been overestimated.

## Conclusions

The use of LTC service varies according to location; care of the elderly in rural areas and the LTC of disabled elderly impose substantial burdens on their families. Our study also revealed that informal care provided by relatives of the elderly involves huge opportunity costs for the caregivers. Chinese policymakers need to promote community care and LTC insurance to relieve the burden on families of the disabled elderly, and particular attention should be given to the rural elderly. Further work is required, such as longitudinal studies, in this area to gain a better understanding of the impact of spending on the care of disabled older persons.

## References

[1] Wong YC, Leung J (2012) Long-term Care in China: Issues and Prospects. J Gerontol Soc Work 55: 570-586. PubMed: 22963116. 22963116 10.1080/01634372.2011.650319

[2] Zhou B, Chen K, Wang J, Wang H, Zhang S et al. (2010) Quality of Life and Related Factors in the Older Rural and Urban Chinese Populations in Zhejiang Province. J Appl Gerontol 30: 199-225.

[3] Du M, Jiang H, Tai B, Zhou Y, Wu B et al. (2009) Root caries patterns and risk factors of middle aged and elderly people in China. Community Dent Oral Epidemiol 37: 260-266. doi:10.1111/j.1600-0528.2009.00461.x. PubMed: 19508272. 19508272

[4] Jiménez-Martín S, Prieto CV (2012) The trade-off between formal and informal care in Spain.Eur J Health Econ 13: 461-490. doi:10.1007/s10198-011-0317-z. PubMed: 21584815. 21584815

[5] Beijing KZ (2011) China National Committee on Aging. Press release, The National Study of disability and Functional Limitations of the Elderly. Chinese.

[6] Riewpaiboon A, Riewpaiboon W, Ponsoongnern K, Van den Berg B (2009) Economic valuation of informal care in Asia: a case study of care for disabled stroke survivors in Thailand. Soc Sci Med 69: 648-653. doi:10.1016/j.socscimed.2009.05.033. PubMed: 19573969. 19573969

[7] Paraponaris A, Davin B, Verger P (2012) Formal and informal care for disabled elderly living in the community: an appraisal of French care composition and costs. Eur J Health Econ 13: 327-336. 21400197 10.1007/s10198-011-0305-3

[8] Johar M, Maruyama S (2011) Intergenerational cohabitation in modern Indonesia: filial support and dependence. Health Econ 20: 87-104. doi:10.1002/hec.1708. PubMed: 21274997. 21274997

[9] Giles J, Wang D, Zhao C (2010) Can China's rural elderly count on support from adult children? Implications of rural-to-urban migration. J Populations Aging 3: 183-204. doi:10.1007/s12062-011-9036-6.

[10] Zhang H (2007) Who will care for our parents? Changing boundaries of family and public roles in providing care for the aged in urban China. Care Manage J 8: 39-46. doi:10.1891/152109807780494087. 17491450

[11] Ku LJE, Liu LF, Wen MJ (2013) Trends and determinants of informal and formal caregiving in the community for disabled elderly people in Taiwan. Arch Gerontol Geriatr 56: 370-376. doi:10.1016/j.archger.2012.11.005. PubMed: 23218520. 23218520

[12] Flaherty JH, Liu ML, Ding L, Dong B, Ding Q et al. (2007) China: the aging giant. J Am Geriatr Soc 55: 1295-1300. doi:10.1111/j.1532-5415.2007.01273.x. PubMed: 17661972. 17661972

[13] Feng Z, Liu C, Guan X, Mor V (2012) China's Rapidly Aging Population Creates Policy Challenges In Shaping A Viable Long-Term Care System. Health Aff Millwood 31: 2764-2773. doi:10.1377/hlthaff.2012.0535. PubMed: 23213161. 23213161 PMC3711106

[14] Wu B, Carter MW, Goins RT, Cheng C (2005) Emerging services for community-based long-term care in urban China. J Aging Soc Policy 17: 37-60. doi:10.1300/J031v17n04_03. PubMed: 16380368. 16380368

[15] Junfang W, Biao Z, Weijun Z, Zhang S, Yinyin W et al. (2009) Perceived unmet need for hospitalization service among elderly Chinese people in Zhejiang province. J Public Health 31: 530-540. 10.1093/pubmed/fdp007 19202151

[16] Kim E-Y, Yang B-M (2005) Cost–effectiveness of long-term care services in South Korea. Archives of gerontology and geriatrics 40: 73-83 15531025 10.1016/j.archger.2004.05.007

[17] Jianfeng W, Gao G, Fan Y, Chen L, Liu S et al. (2006) The estimation of sample size in multi-stage sampling and its application in medical survey. Applied mathematics and comuputation 178:239-249.

[18] Ama NO, Seloilwe ES (2010) Estimating the cost of care giving on caregivers for people living with HIV and AIDS in Botswana: a cross-sectional study. Journal of The International AIDS Society 13: 14. 20406455 10.1186/1758-2652-13-14 PMC2880016

[19] Mullahy J (1998) Much ado about two: reconsidering retransformation and the two-part model in health econometrics. J Health Econ 17: 247-281. doi:10.1016/S0167-6296(98)00030-7. PubMed: 10180918. 10180918

[20] Buntin MB, Zaslavsky AM (2004) Too much ado about two-part models and transformation? J Health Econ 23: 525-542. doi:10.1016/j.jhealeco.2003.10.005. PubMed: 15120469. 15120469

[21] Schwarzkopf L, Menn P, Leidl R, Wunder S, Mehlig H et al. (2012) Excess costs of dementia disorders and the role of age and gender - an analysis of German health and long-term care insurance claims data. BMC Health Serv Res 12: 165. doi:10.1186/1472-6963-12-165. PubMed: 22713212. 22713212 PMC3425320

[22] Wu B, Mao ZF, Zhong RY (2009) Long-Term Care Arrangements in Rural China: Review of Recent Developments. J Am Med Directors Assoc 10: 472-477. doi:10.1016/j.jamda.2009.07.008. 19716063

[23] Zhu CW, Torgan R, Scarmeas N, Albert M, Brandt J et al. (2008) Home Health and Informal Care Utilization and Costs Over Time in Alzheimer's Disease. Home Health Care Serv Q 27: 1-20. doi:10.1300/J027v27n01_01. PubMed: 18510196. 18510196 PMC2631654

[24] Liu J, Chi I, Chen G, Song X, Zheng X (2009) Prevalence and correlates of functional disability in Chinese older adults. Geriatr Gerontol Int 9: 253-261. doi:10.1111/j.1447-0594.2009.00529.x. PubMed: 19702935. 19702935

[25] LaPlante MP, Harrington C, Kang T (2002) Estimating paid and unpaid hours of personal assistance services in activities of daily living provided to adults living at home. Health Serv Res 37: 397-415. doi:10.1111/1475-6773.029. PubMed: 12036000. 12036000 PMC1430364

[26] Shaw C, McNamara R, Abrams K, Cannings-John R, Hood K et al. (2009) Systematic review of respite care in the frail elderly. Health Technol Assess 13. 10.3310/hta13200 19393135

[27] Hellström Y, Hallberg IR (2004) Determinants and characteristics of help provision for elderly people living at home and in relation to quality of life. Scand J Caring Sci 18: 387-395. doi:10.1111/j.1471-6712.2004.00291.x. PubMed: 15598246. 15598246

[28] Ku L-JE, Liu L-F, Wen M-J (2012) Trends and determinants of informal and formal caregiving in the community for disabled elderly people in Taiwan. Arch Gerontol Geriatr. 10.1016/j.archger.2012.11.005 23218520

[29] de Meijer C, Koopmanschap M, d’ Uva TB, van Doorslaer E (2011) Determinants of long-term care spending: Age, time to death or disability? J Health Econ 30: 425-438. doi:10.1016/j.jhealeco.2010.12.010. PubMed: 21295364. 21295364

[30] Chen F, Liu G (2009) Population aging in China. International handbook of population aging. Springer Verlag pp. 157-172.

